# Maternal Selenium and Developmental Programming

**DOI:** 10.3390/antiox8050145

**Published:** 2019-05-25

**Authors:** Athanasios C. Pappas, Evangelos Zoidis, Stella E. Chadio

**Affiliations:** 1 Department of Nutritional Physiology and Feeding, Faculty of Animal Science, Agricultural University of Athens, 75 Iera Odos, 11855 Athens, Greece; apappas@aua.gr (A.C.P.); ezoidis@aua.gr (E.Z.); 2 Department of Anatomy and Physiology of Domestic Animals, Faculty of Animal Science, Agricultural University of Athens, 11855 Athens, Greece

**Keywords:** embryonic development, maternal nutrition, periconceptional period, progeny, selenium, selenoproteins

## Abstract

Selenium (Se) is an essential trace element of fundamental importance to health due to its antioxidant, anti-inflammatory, and chemopreventive properties, attributed to its presence within at least 25 selenoproteins (Sel). In this review, we describe some of the recent progress, in our understanding, on the impact of maternal Se intake during the periconceptional period on offspring development and health. Maternal nutrition affects the performance and health of the progeny, and both maternal and offspring Se supplementations are essential for the optimal health and antioxidant protection of the offspring. The case of Se in epigenetic programming and early life nutrition is also discussed.

## 1. Introduction

Maternal nutrition is of critical importance for fetal growth. An unbalanced nutrient supply will result in low birthweight and, subsequently, compromise future growth and development. Apart from short-term outcomes, accumulating evidence from animal and human studies indicates that early life experiences can impact offspring phenotype, a concept known as Developmental Origins of Health and Disease (DOHaD) hypothesis [1]. Variation in the maternal plane of nutrition, and particularly maternal under or over nutrition, appears to be a dominant factor in developmental programming, in both humans and livestock, including metabolic [2], productive [3,4], and reproductive outcomes [5,6]. In addition, maternal nutritional imbalances in terms of both macro and micro nutrients can induce oxidative stress, which may affect fetal growth and development [7]. Recently, the role of colostrum in affecting the critical developmental process, as a conduit of transmission of certain bioactive molecules from mother to offspring, has attracted considerable attention, according to lactocrine signaling hypothesis [8]. The periconceptional period is playing a crucial role in programming effects, since it is characterized by extensive reorganization of cellular phenotype during oocyte maturation, fertilization, and embryonic genome activation [9]. Even in poultry species, the prenatal environment can be divided into pre-lay and egg storage/incubation environments, both of which can affect offspring outcomes. In particular, maternal nutrition is of paramount importance because all nutrients required by the developing embryo are deposited in the egg and therefore exert an effect not only during embryonic but also during posthatch development [10]. 

Apart from the well-established role of micronutrients in short-term pregnancy outcomes [11], accumulating evidence also supports a role for micronutrients in developmental programming [12,13,14]. Trace elements affect the endocrine regulation of energy metabolism and energy homeostasis, as well as oxidative balance, both of which are related to normal growth [15]. In particular, Se possesses antioxidant, chemopreventive, and anti-inflammatory properties and is considered as a trace element of great importance to the health of both mammals and avian species. Its action is related to its presence within at least 25 selenoproteins, i.e., Se-containing proteins products of twenty-five genes. Among them some are well characterized with respect to their function like the glutathione peroxidases (GPX1, GPX2, GPX3, GPX4, and GPX 6), the thioredoxin reductases (TXNRD1, TXNRD2, and TXNRD3), and the iodothyronine deiodinases (DIO1, DIO2, and DIO3). Other selenoproteins include but are not limited to selenophosphate synthetase 2 (SEPHS2), selenoprotein F or selenoprotein 15 (SELENOF), selenoprotein H (SELENOH), selenoprotein I (SELENOI), selenoprotein K (SELENOK), selenoprotein M (SELENOM), selenoprotein N (SELENON), selenoprotein O (SELENOO), selenoprotein P (SELENOP), selenoprotein S (SELENOS), selenoprotein T (SELENOT), selenoprotein V (SELENOV), and selenoprotein W (SELENOW) [16,17]. Selenoprotein P is the major Se transporting protein [18], and is considered the only known protein that contains multiple selenocysteine residues per protein molecule [19]. 

Selenomethionine and selenocysteine are identical to methionine and cysteine, respectively, except that the sulphur atom is replaced by Se. Plants synthesize selenomethionine and a variety of methylated amino acids. Plants absorb Se from the soil in the form of selenite or selenate and synthesize selenomethionine [20,21]. In feed ingredients, Se can be found in form of selenomethionine. In addition, to the amount of Se received from feed ingredients, feeds of farm animals are widely supplemented. Selenium is generally supplemented in the form of inorganic Se or in organic form, and the most widely used sources include sodium selenite, sodium selenate, controlled-release sodium selenite bolus, SeMet, zinc L-selenomethionine complex, hydroxy-analogue of selenomethionine, Se-yeast, elemental Se at nano size, soybean Se proteinate, Se-enriched malt, chlorella algae, cabbage, and garlic [22,23,24,25]. Selenium efficiency depends on the level of supplementation and form of Se in the diet, and organic sources proven to be effective sources of Se for poultry and animal production [26,27]. In EU, the maximum supplementation with organic Se is 0.2 mg Se per kg of complete feed, while the maximum content of Se (total Se) is 0.5 mg/kg of complete feed [28]. Similarly, the Food and Drug Administration (FDA) regulations limit the amount of dietary Se supplementation, to 0.3 mg/kg (as fed) [29]. Given the FDA limits, any concentration that exceeds 0.3 mg/kg and is below the maximum tolerable level can be considered as supranutritional. 

Recently, the role of Se in maternal nutrition and its impact on the Se status of offspring has received considerable attention. In mammals, Se is transferred both via the placenta, the colostrum, and milk to the fetus and the neonate [30]. In avian species, laying hen transfers Se to the egg and in turn to the developing embryo and newly hatched chick [10,31,32]. Selenium affects non-enzymatic and enzymatic antioxidant defense mechanisms, helping build a strong antioxidant defense for both mother and the developing embryo. Recent studies in humans have also revealed an association between maternal Se status and specific early childhood outcomes. Both low and high levels of cord serum Se have adverse effects on an infant’s neurobehavioral development [33]. More interestingly, a positive effect of maternal selenium status on children’s development at 1.5 years of age has been reported, related to language and psychomotor development improvement [34]. These findings have been supported by data from human cohort studies. In particular, in a Polish cohort study involving 410 mother-child pairs, a significant positive association was detected between Se levels in the blood collected during the first trimester of pregnancy and child motor skills at 1 and 2 years of age and cognitive development at 2 years of age [35].

## 2. Combined Effects of Maternal Plane of Nutrition and Selenium Status on Progeny

Numerous studies have investigated the interaction between maternal plane of nutrition and Se status on a number of physiological parameters in the offspring. In particular, inconsistent results of the effects of maternal supranutritional Se supplementation regarding birth weight or weight at weaning have been reported in sheep ranging from no effect [36] to an increase in birth weight and weight at weaning, regardless of global maternal nutrient intakes [37,38]. On the other hand, supranutritional Se supplementation to nutritionally restricted dams (60% of their needs) resulted in improved fetal growth, implicating a sparing effect of Se and long-term developmental consequences [37,38].

In pigs, maternal supplementation with SeMet significantly increases litter weight at weaning and body mass of piglets [39], whereas in chickens, inclusion of Se in the maternal diet could positively affect embryo viability, hatchability, and growth of the progeny [10,31,32]. In the same species, dietary inclusion of organic Se (zinc L-selenomethionine complex) promoted heavier hatchling weight until egg production peak, (33 week), without affecting hatchling quality [40]. Moreover, supplementation of the breeder’s diet with organo-Se appears to enhance the DHA concentration of the chick brain [10], in good agreement with the well accepted role of Se in protecting polyunsaturated fatty acids (PUFA) oxidation [41]. 

In large animals, the majority of the studies examining the role of Se in modifying the effects of nutrition are mostly limited to the late gestation fetus, and only a few examine later outcomes. Six-months old lambs, derived from ewes supplemented with supranutritional Se and artificially reared to avoid confounding effects with colostral Se, showed greater small intestinal weight, which, however, was not accompanied by an increase in jejunal cell proliferation [42]. As is pointed out by authors, this increase could be translated to increased crypt cell proliferation and altered villi number, leading probably to more efficient nutrient absorption.

It has been well documented that maternal nutritional status impacts mammary gland development and, subsequently, colostrum and milk yield [36,43]. However, data from primiparous ewes indicate that supranutritional supplementation from conception to term may be a more potent factor in driving milk production [43]. Interestingly, Se supplementation in ewes at approximately 10 times above current recommendations was reported to increase mammary gland capillary area density and vascularity, a finding explaining the increase in milk production, possibly by enhancing the nutrient availability to the mammary gland [44]. 

Recently, the role of Se in metabolic programming has gained interest [45]. This micronutrient, through the activity of selenoproteins, such as glutathione peroxidase and selenoprotein P, has been reported to play a role in the regulation of enzymes in the insulin signaling cascade, the expression of lipogenic enzymes, and carbohydrate metabolism in the liver [46,47,48]. In this respect, studies in sheep revealed an interaction between nutritional and Se maternal status on insulin sensitivity in offspring. Lambs born to high-Se but restricted-diet mothers exhibit an elevated insulin release following intravenous glucose tolerance test [37]. Moreover, the same offspring were characterized by greater total visceral adiposity, attributed mainly to an increase in visceral rather than omental fat [37]. The involvement of Se and its selenoproteins, glutathione peroxidase and selenoprotein P, in insulin regulation has been confirmed from a number of studies, indicating a role in insulin signaling cascade [48,49]. However, recent evidence indicates that effects are related to the level of supplementation. Adequate concentrations exert a key role in insulin function, but an excess is associated with insulin resistance [50]. The underlying mechanism probably involves formation of antioxidant selenoproteins and thus reduced concentrations of reactive oxygen species, which are required for insulin signaling [51,52]. Therefore, the exact impact of maternal Se and especially the level of supplementation in insulin-glucose axis regulation in the offspring is not yet clear and needs further elucidation. A summary of selected animal studies reporting combined effects of maternal plane of nutrition and Se status on progeny is presented in Table 1.

## 3. Effects of Maternal Selenium Status on Immunity of the Offspring 

The important role of Se in innate and adaptive immune response has been attributed to the regulation of oxidative stress and redox mechanisms via the action of selenoproteins [53]. In particular, Se is known to play a role in immunocompetence through its incorporation into glutathione peroxidases and thioredoxin reductase [54]. A role of Se in enhancing offspring passive immunity through colostrum tranfer of immunoglobulins is well documented [55,56]. However, the source and level of supplementation in maternal diet is still debatable. Data from ewes showed an effect of dose and source of supplementation on IgG transfer to lambs with optimal dose for maximal transfer being different between Na selenite and Se-yeast [57]. Calves born to dams supplemented with Se yeast for the last 8 weeks of pregnancy showed increased serum-IgG concentrations for the first 14 days, which persisted up to 60 days of age [58]. These effects of increased absorption efficiency may be mediated by either activating or prolonging pinocytosis processes [59]. 

The effect of Se status on health of calves is postulated not to be limited only to effects on neonatal caves mediated by colostral transfer of maternal immunoglobulins. In this context, Yoshitaka et al. [60] reported non-specific as well as specific immunity parameters in five weeks old calves originating from cows fed a basal diet or cows supplemented with Se. Offspring from supplemented mothers showed an earlier neutrophil oxidative reaction, associated with phagocytosis and a higher stimulation of lymphocytes, following concavalin simulation. It is possible that Se-deficient animals may have defective function of neutrophils due to lack of glutathione peroxidase activity [61]. Future studies will further enlighten the benefits of applying supranutritional Se supplementation in maternal diets in order to enhance passive immunity and boost the immunity of the progeny.

On the other hand, as the immune system develops, predominantly represents a potential target for fetal programming. Therefore, perturbations to the emerging immune system resulting from early macro and micro nutritional imbalances may have not only immediate consequences for susceptibility to infection after birth but also implications for later life function of the immune system [62,63]. Rat pups derived from Se-deficient mothers show impaired thymocyte activation in vitro and a decrease in the percentages of CD8 cytotoxic T-lymphocytes, CD2 T-lymphocytes, B-lymphocytes, and natural killer cells in their spleen [64].

The source of Se has been reported to affect the immunity of the offspring by altering the relative expression of a number of lymphocyte cytokines [65]. In particular, foals born to mares supplemented with organic Se showed an increase expression of IL 2 and interferon gamma, important for lymphocyte proliferation and cellular immunity, respectively, as well as decreased expression of the proinflammatory cytokine tumor necrosis factor alpha (TNF-α). There is growing evidence that Se can affect gene expression on both transcriptional and translational levels [66,67,68], further supporting its programming effects. A summary of selected animal studies reporting effects of maternal Se status on immunity of the offspring is presented in Table 2.

## 4. Effects of Maternal Selenium Status on Reproduction of the Offspring 

The reproductive axis and its hormonal control systems are largely established during fetal life, representing a target for developmental programming [69,70]. Accumulating evidence supports a crucial role of Se for fetal reproductive organ development in both sexes, probably mediated by its antioxidant properties. In particular, the testis represents a specific target for Se function, as being crucial for normal spermatogenesis and maintenance of male fertility [71].

Recent data from a number of studies in male goat offspring support a role of maternal Se status in the development of testis and spermatogenesis after weaning. Supplementation of maternal diet with Se (as Se enriched yeast) at different levels (0, 0.5, 2.0, and 4.0 mg/kg) improved the testosterone level and promoted the expression of testosterone related genes, in offspring testis, which, however, was decreasing as Se supplementation was increased [72]. Moreover, offspring exhibited different expression profile for a number of germ cycle-related genes in their testis, depending on the different supplementation levels. In particular, it was shown that both maternal Se deficiency and excess could prevent the completion of cell cycle and induce an increase in the apoptotic rate of germ cells [73]. These data indicate that supplementation of maternal diet during gestation could provide the Se necessary for normal development of reproductive function of the offspring, but on the other hand, underline the importance of determining the necessary level of supplementation, as Se excess leads to opposite effects.

Regarding female organ development, Grazul Bilska et al. [74] reported that maternal Se dietary intake may be involved in the regulation of early folliculogenesis and cellular proliferation of the follicles, blood vessels, and stromal tissues of fetal ovaries in sheep. In support of these findings, in vitro studies with bovine granulosa cells revealed a positive action of Se in promoting granulosa cell proliferation and E2 production, an effect possibly mediated through decreased NO production caused by Se [75].

In avian species, similar to mammals, reproduction outcome is greatly affected by the quality of the semen of the male. Avian semen is rich in PUFA and thus susceptible to lipid peroxidation. In detail, phospholipids of chicken spermatozoa are rich in long-chain fatty acids of the n-6 family, most notably arachidonic (20:4n−6) and docosatetraenoic (22:4n−6) acids [76]. In duck, spermatozoa contain a much higher proportion of docosahexaenoic acid (22:6n-3) in the sperm lipids [77], indicating the susceptibility to peroxidation. Optimal Se status of parent stock (breeders) can maintain the semen quality and provide the antioxidant environment for the developing embryo [26]. In this context, the average Se concentration in the semen of chickens fed a non-supplemented diet (0.1 mg/kg) was shown to be 47 ± 3 ng/g, while in breeders fed a Se supplemented diet (0.5 mg/kg) it was shown to be 101 ± 10 ng/g [78]. Regarding the link between female reproduction and Se, a preliminary study on broiler breeders established a positive relationship between Se addition and gene transcription [79]. In detail, Se was associated with genes encoding energy-associated mitochondrial proteins and protein synthesis networks, indicating that Se, especially in the organic form, is necessary for optimal energy production and protein translation in female reproductive tissues [79]. Yuan et al. [80] reported little influence on the egg production rate and fertilization rate of broiler breeders fed Se from different sources. However, organic Se sources at 0.3 ppm resulted in greater deposition of Se in breeders, egg, embryo, and offspring chicks compared to inorganic Se. The same authors reported that among organic Se sources, SeMet had higher tissue Se retention than Se yeast, indicating that maternal effects may be more pronounced when broiler breeders are fed with SeMet [80].

One of the significant gaps regarding the role of maternal Se on reproductive health is the identification of the best timing of supplementation. Recently, the periconceptional period has attracted more attention in the context of developmental programming concept, being the potentially more critical period in which developmental plasticity is vulnerable to environmental challenges, including maternal macro and micro nutrition. This period encompasses gametogenesis, fertilization, conceptus formation, implantation, and placentation, which represent particular time windows during which epigenetic changes can have long-lasting consequences on the development and function of the tissues and therefore long-term effects on offspring phenotype [81]. An experiment in mice, involving Se supplementation during three different stages of periconception period, revealed that the pregestation and gestation periods are optimal developmental windows of Se supplementation for improved quality of blastocysts and reduced preimplantation loss [82].

It is well acknowledged that gametes and embryos function better when the redox potential is higher, but on the other hand it must be kept in mind that excess supplementation of antioxidants may suppress the beneficial signaling function of reactive oxygen species and impair their reproductive potential [83].

## 5. Conclusions

Selenium, in most cases at supranutritional levels, may have positive effects during gestation, particularly in situations during which animals are metabolically or nutritionally stressed. Selenium added on both prenatal and postnatal diets may provide an effective antioxidant system. More interestingly, the findings presented in this review contribute to the current concept that maternal nutrition and micronutrient status can potentially impact offspring health and performance. However, the mechanisms underlying these effects are still unidentified. Current evidence clearly indicate that Se controls the expression levels of both selenoproteins and non-selenoproteins genes [84]. In this context, previous studies in mice have shown that low intake of Se, downregulated genes involved in detoxification, and upregulated genes involved in DNA damage and oxidative stress [85], while progeny from breeders fed relative high level of vitamins and trace minerals, including Se, have shown higher expression of intestinal genes that affect the turnover and proliferation of intestinal cells compared to chicks originating from breeders fed a diet low in vitamins and trace elements [86]. More recently, the role of maternal Se in increasing protein synthesis and SELENOW mRNA levels as well as the expression of a number of genes related to muscle growth has been reported for poultry offspring [87]. Epigenetic modulation of gene transcription provides a possible mechanism through which maternal Se supplementation can alter gene expression in the developing fetus leading to permanent effects in later life. Investigation of the role of Se in epigenetic modulation is still in its infancy, and it is closely related to human health outcomes [88]. Recent data suggest critical roles of certain selenoproteins in the epigenetic regulation of promoter methylation, histone modifications, and noncoding RNA expressions [89].

Further research is needed to fully elucidate the role of maternal Se on offspring health and productivity in animals and to optimize maternal Se recommendation. There is also need for robust and extended studies, which will enable us to better define the prerequisites under which offspring may benefit from maternal selenium supplementation.

## Figures and Tables

**Table 1:** A summary of selected studies describing the combined effects of maternal plane of nutrition and selenium status on progeny.

Animal Model	Study Design	Key Findings as Reported by Authors	Reference
Broiler breeders/broilers	Low (0.1 mg/kg) or High (0.5 mg/kg) selenium (Se) status (Se yeast) and soyabean oil and low or high (0.5 mg/kg) Se status and fish oil and progeny fed high or low (20% less energy and protein) diets with similar Se content	Broilers fed for two weeks posthatch a diet with similar Se content but hatched from parents fed high-Se diets had higher tissue Se concentrations than those hatched from parents fed diets low in Se Supplementation of the maternal diet of chicks with organo Se compounds enhanced the concentration of docosahexaenoic acid in the brain of progeny	[10]
Broiler breeders/broilers	Vitamin E at two levels (30, 120 mg/kg) and two sources of Se (Sodium selenite or Zinc-L-SeMet) at 0.4 mg/kg	Inclusion of organic selenium in breeders diet led to heavier hatchling weight until egg production peak (33 wk) Hatchability of the eggs from 29 wk old breeders fed 120 mg vitamin E/kg feed was higher than that of breeders fed 30 mg vitamin E	[40]
Ewes/lambs	Adequate (9.5 μg/kg body weight (BW)) or High (81.8 μg/kg BW) and nutritional status 60% of metabolisable energy requirements (Restricted), 100% (Control), and 140% (High)	Female lambs from high Se ewes were heavier at birth Maternal Se intake can enhance fat deposition in female offspring Nutritional intake × Se status interaction on growth rate of lambs and insulin response on a glucose tolerance test; thus, both maternal nutritional level and Se intake can influence insulin sensitivity	[37]
Ewes/lambs	Adequate (9.5 μg/kg BW) or High (81.8 μg/kg BW) and nutritional status 60% of metabolisable energy requirements (Restricted), 100% (Control), and 140% (High)	High maternal Se led to greater jejunal capillary area density in the offspring	[42]
Ewes/lambs	Adequate (9.5 μg/kg BW) or High (81.8 μg/kg BW) and nutritional status 60% of metabolisable energy requirements (Restricted), 100% (Control), and 140% (High)	Colostrum and milk yield greater in high- vs. adequate-Se-fed ewes	[43]
Ewes/lambs	Adequate (9.5 μg/kg BW) or High (81.8 μg/kg BW) and nutritional status 60% of metabolisable energy requirements (Restricted), 100% (Control), and 140% (High)	Progeny from high-Se ewes had greater capillary surface density compared with those from adequate group	[44]
Sows/piglets	0.042 mg/kg or 0.3 mg/kg as sodium selenite or selenomethionine	Maternal selenomethionine vs. inorganic Se significantly increased the weaning litter weight and average weight of piglets	[39]
Dam rats/pups	0.01 mg Se/kg diet (deficient-low), 0.1 mg /kg, and 0.5 mg Se/kg diet (supplemented-high)	Pups born from mothers fed excess Se exhibited insulin resistance	[50]

**Table 2:** A summary of selected animal studies reporting effects of maternal Se status on immunity of the offspring.

Animal Model	Study Design	Key Findings as Reported by Authors	Reference
Sows/piglets	Sows during late gestation and lactation fed dietary Se level low (0.3 mg/kg) or high (1.2 mg/kg) and subjected to heat stress or not	Colostrum and milk composition from sows fed increasing Se was improved compared to that from low-Se-fed sows Increasing Se intake of sows led to improved piglet preweaning survival, immunoglobulin transfer, and better antioxidant status	[56]
Ewes/lambs	No Se or adequate (4.9 mg Se/wk) or two supranutritional levels (14.7 and 24.5 mg Se/wk) from inorganic or organic sources	Ewes during pregnancy fed supranutritional Se supplementation had increased colostral IgG concentrations Optimal supplementation rate for IgG transfer from ewe to lamb may differ between inorganic and organic sources	[57]
Beef cows/calves	Control cows were fed non-Se-fortified alfalfa hay (5.3 mg Se/head daily plus inorganic Se 3 mg Se/head daily). Medium-Se cows were fed Se-fortified alfalfa hay (27.6 mg Se/head daily) and high-Se cows were fed Se-fortified alfalfa hay (57.5 mg Se/head daily)	Using ovalbumin as a model to examine passive transfer, it was reported that calves born from high-Se cows given of age at 12 h of age an oral dose of ovalbumin had higher serum ovalbumin concentrations across the first 48 h of life compared to calves born from control cows	[55]
Dairy dam/calves	Basal maternal diet with 0.3 mg Se/kg dry matter (DM) plus 0 or 105 mg Se/wk as yeast	Increased serum IgG concentrations persisted up to 60 d of age	[58]
Beef dam/calves	Control and Se-enriched fed dams (Se: 0.525 mg/100 kg of body weight/day)	After three weeks, calves of dams fed Se-supplemented diets had higher serum-Se concentration than in those of control dams Suckling calves had enhanced neutrophil and lymphocyte functions	[60]
Mare/foals	Deficient (0.05 mg Se/kg DM), inorganic with 0.3 mg Se/kg DM, and organic as yeast with 0.3 mg Se/kg DM	Foals from organic group had higher gene expression of interferon gamma and interleukin-2 and lower tumor necrosis factor alpha expression compared with foals in the inorganic group	[65]
